# INTRAINDIVIDUAL VARIABILITY IN SLEEP DURATION IS ASSOCIATED WITH HIPPOCAMPAL VOLUME IN MID LIFE

**DOI:** 10.1093/geroni/igad104.1814

**Published:** 2023-12-21

**Authors:** Jose Diaz, David Almeida

**Affiliations:** Penn State University, University Park, Pennsylvania, United States; The Pennsylvania State University, State College, Pennsylvania, United States

## Abstract

Sleep is associated with global cognition, consolidation of memories, and structural & functional integrity of areas of the brain that support healthy cognition. While there has been research on sleep in older adults that indicates associations between poor sleep, cognitive decline, memory performance, and changes in the brain, there has been comparatively little work assessing the impact of sleep on the structure of the brain during middle adulthood. Changes in the brain begin much earlier than older adulthood, with midlife seeing cortical thinning and a decrease in brain volume in areas that support healthy cognitive functioning and memory, such as the hippocampus. Understanding how sleep may contribute to hippocampal volume in midlife may help inform how changing poor sleep is important for supporting successful aging. The current study examines the associations between 7 days of actigraphy measured sleep and hippocampal volume (MRI) in 82 midlife adults (Mage = 49.13 years, SD = 11.74; 28-76 years old) who completed the Biomarker and Neuroscience projects in the Midlife in the United States Refresher study. Results indicated that bi-lateral hippocampal volume was negatively associated with high (vs low) intraindividual total sleep time variability (>= 60 min; mean average deviation), with a 280 and 252 mm3 (p’s = .006 & .035) volume reduction for left and right hemispheres respectively. This relationship was not seen for other measures of sleep quality and the analysis additionally controlled for age, sex, BMI, race, depression status, education, and subjective physical & mental/emotional health.

